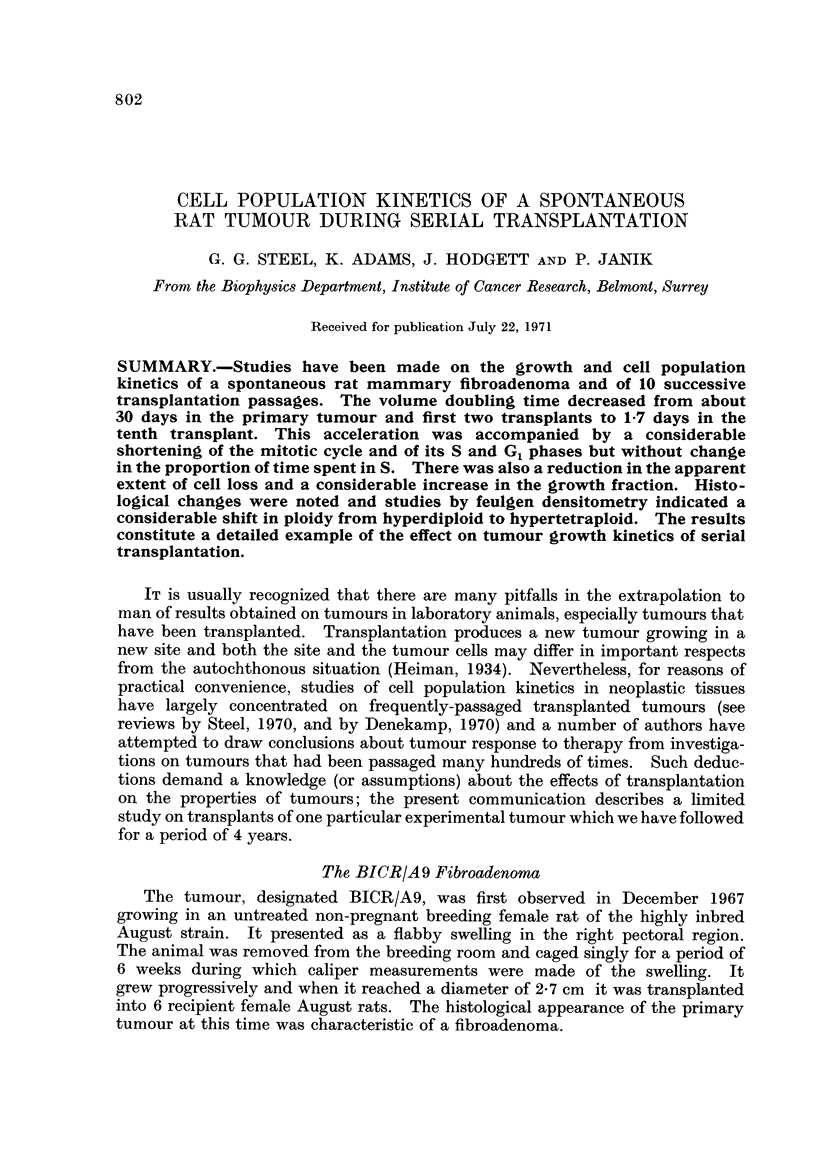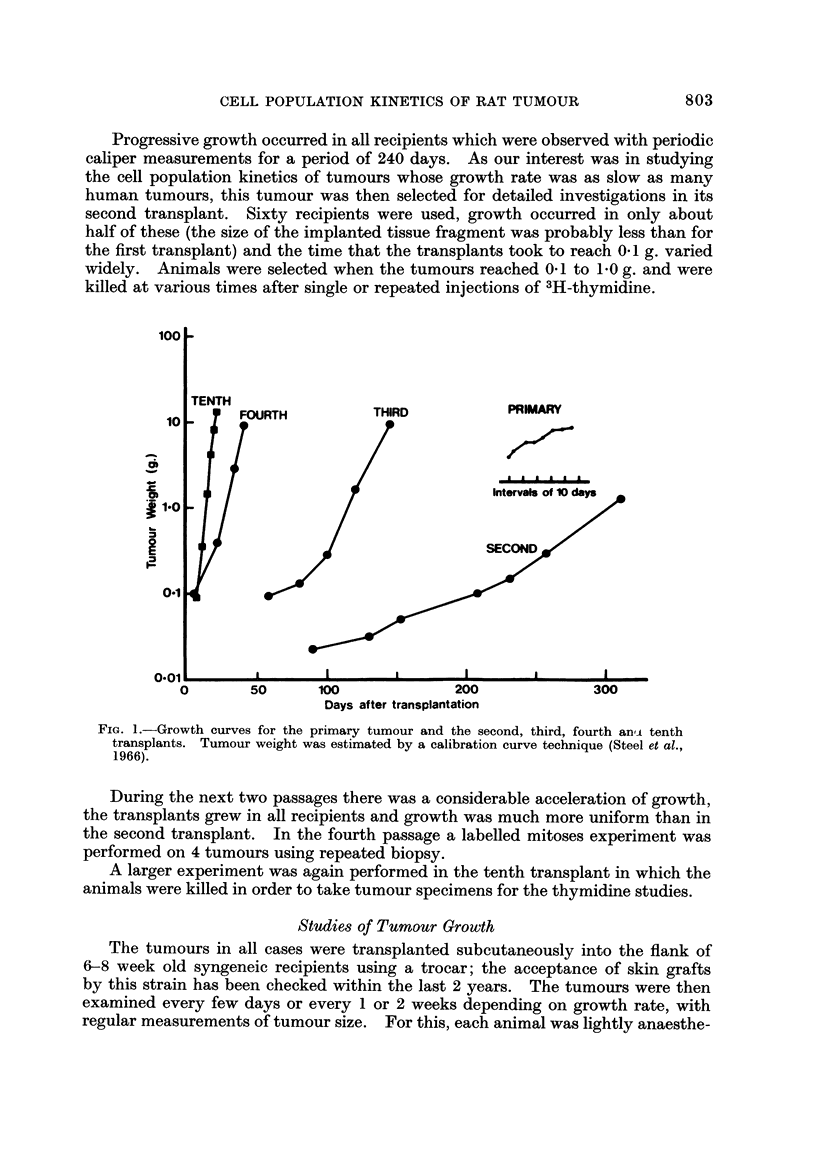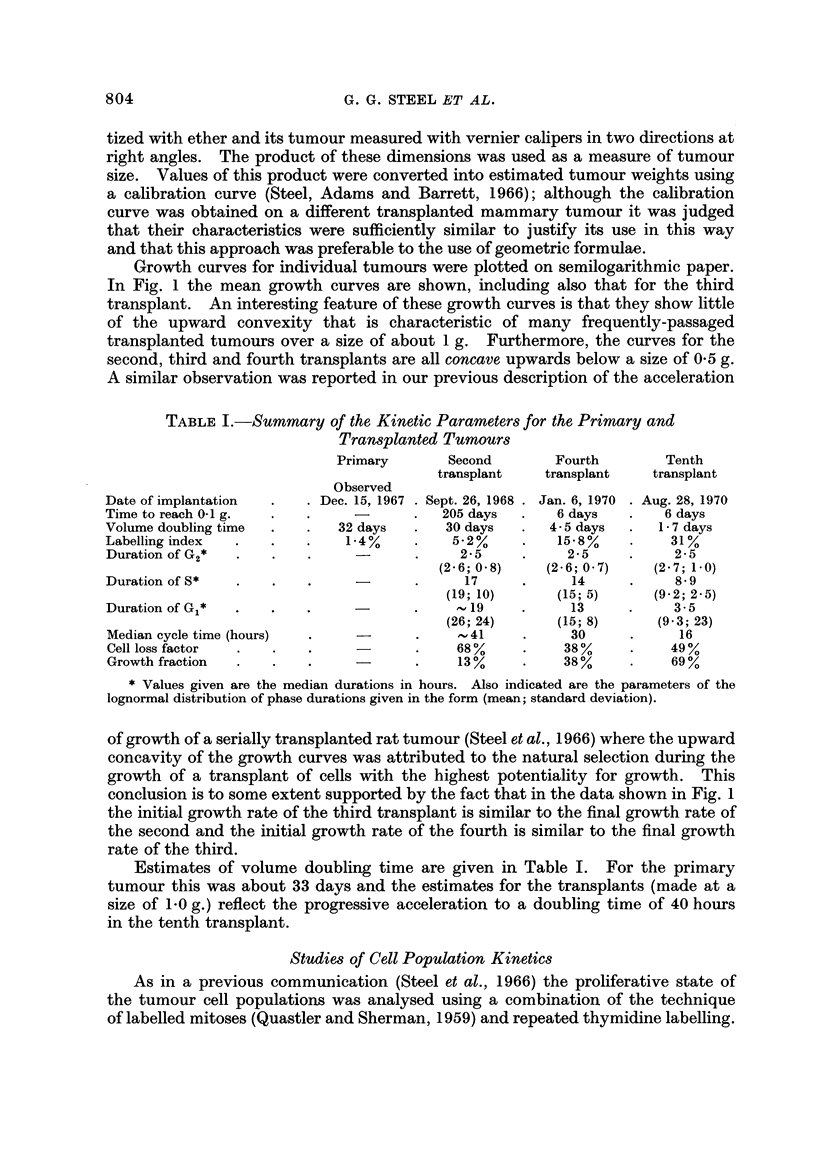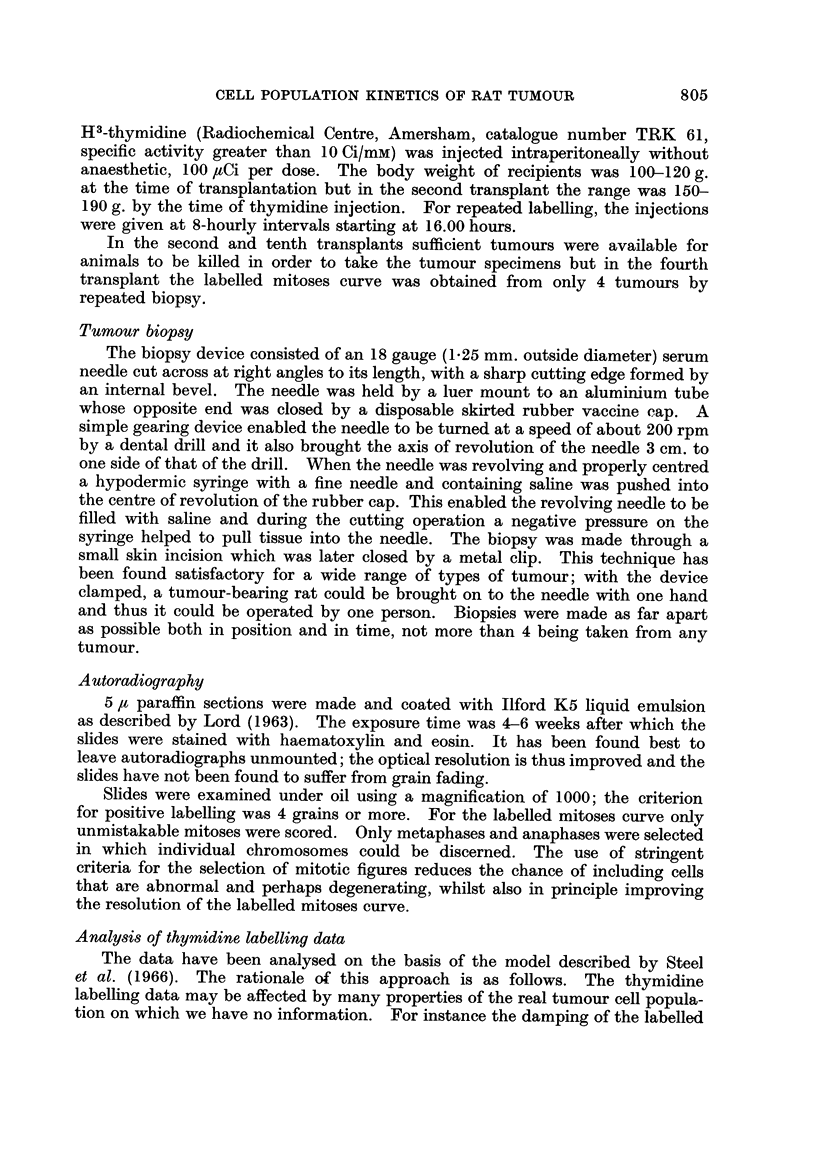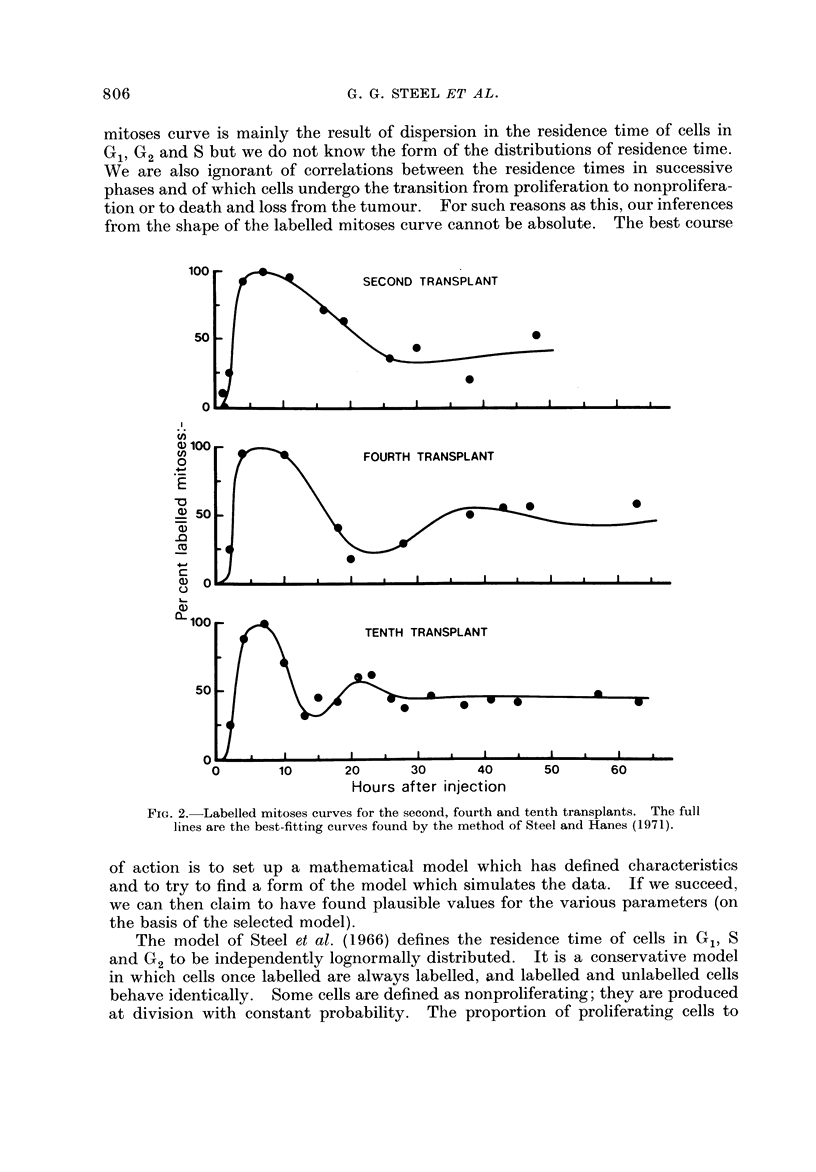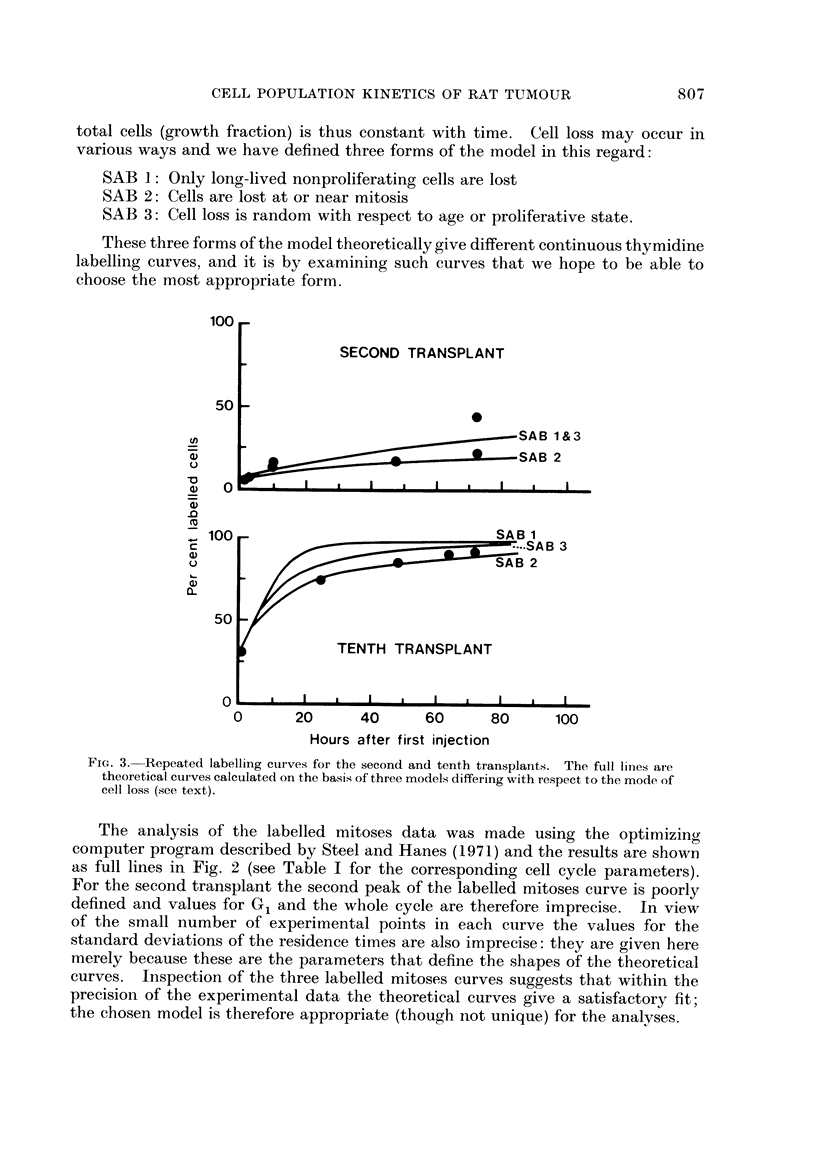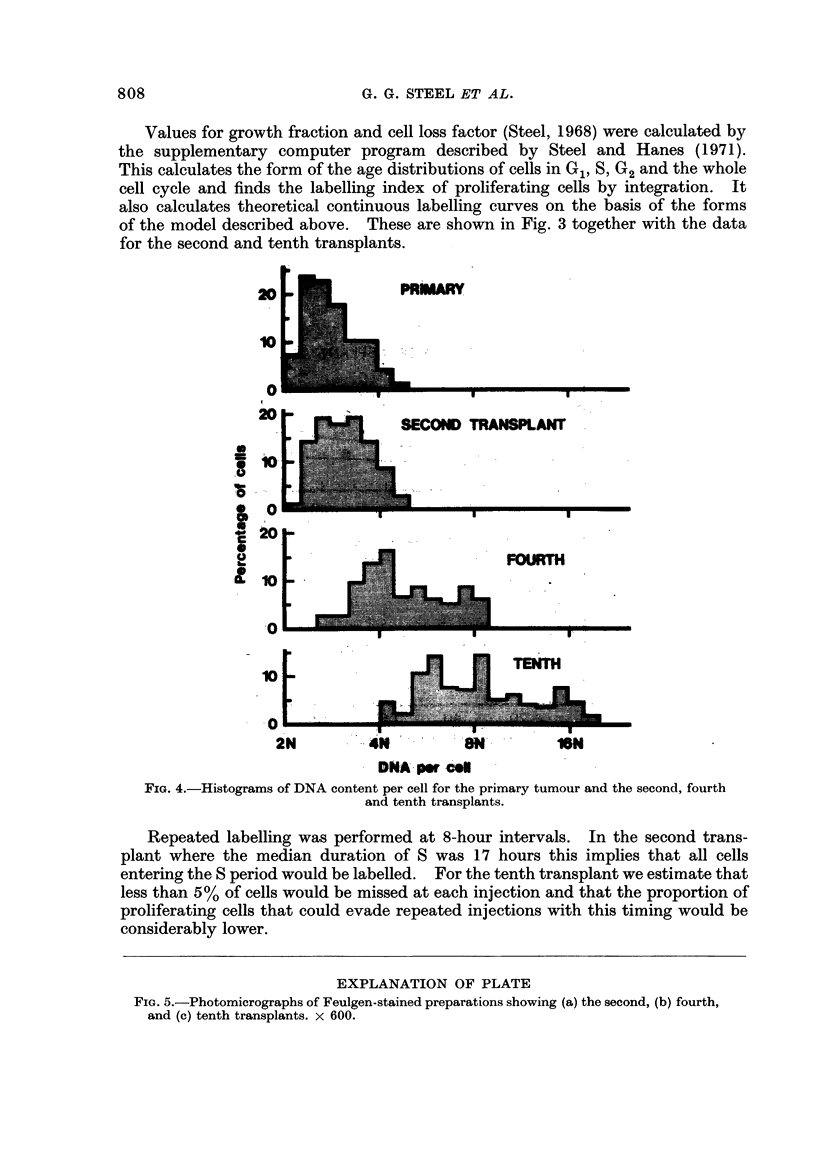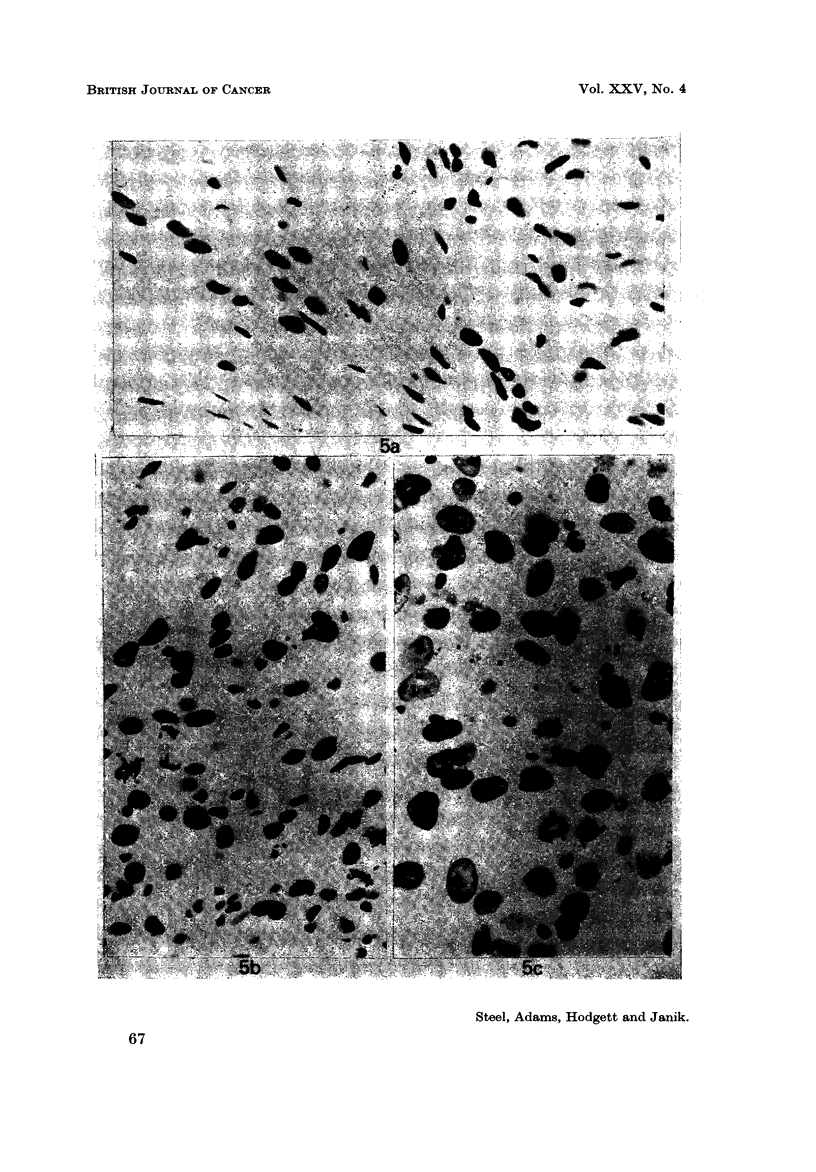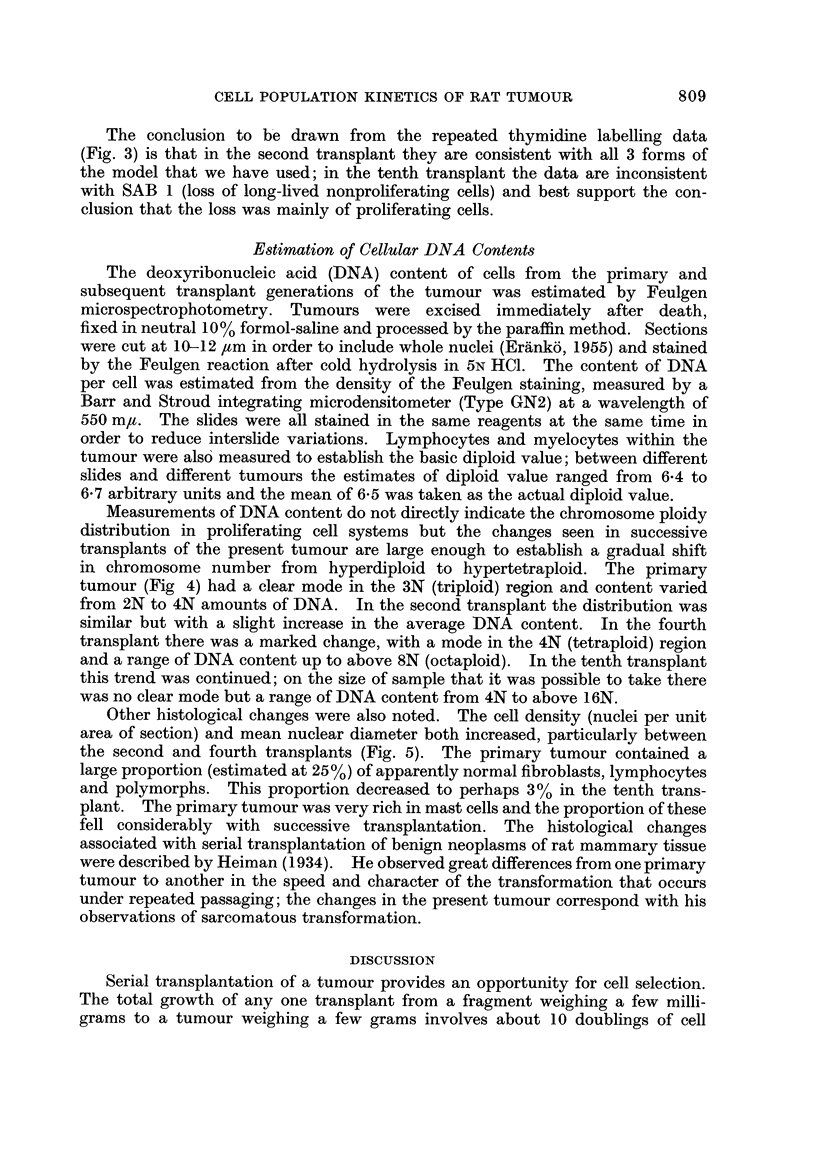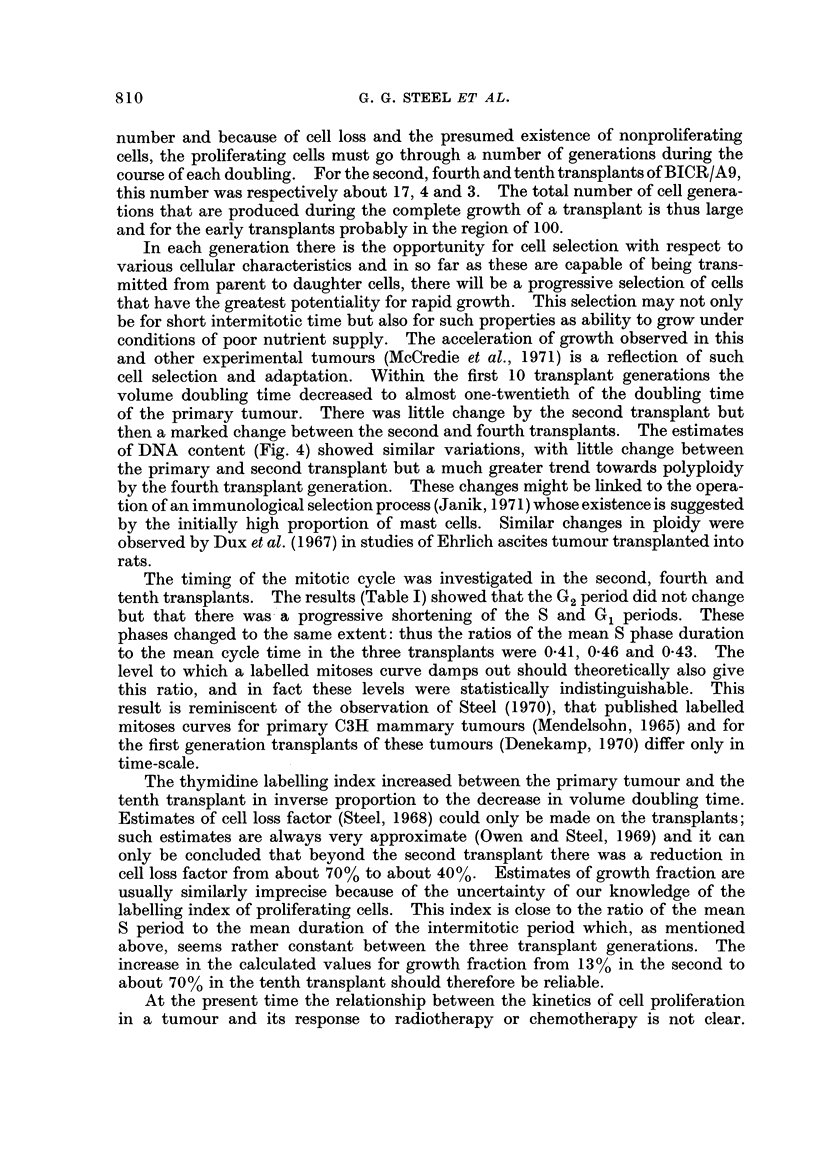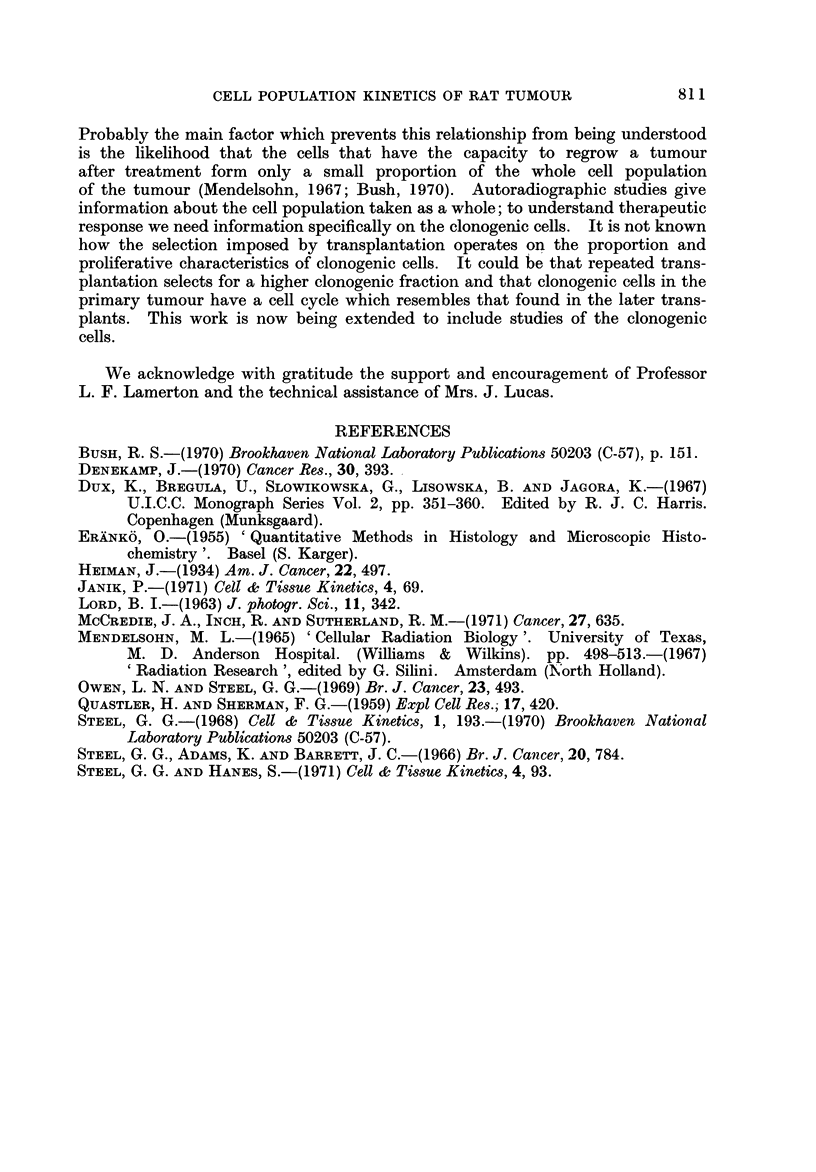# Cell Population Kinetics of a Spontaneous Rat Tumour During Serial Transplantation

**DOI:** 10.1038/bjc.1971.94

**Published:** 1971-12

**Authors:** G. G. Steel, K. Adams, J. Hodgett, P. Janik

## Abstract

**Images:**


### CELL POPULATION KINETICS OF A SPONTANEOUS RAT TUMOUR DURING SERIAL TRANSPLANTATION

G. G. STEEL, K. ADAMS, J. HODGETT AND P. JANIK

*From the Biophysics Department, Institute of Cancer Research, Belmont, Surrey*

Received for publication July 22, 1971

**SUMMARY.—Studies have been made on the growth and cell population kinetics of a spontaneous rat mammary fibroadenoma and of 10 successive transplantation passages. The volume doubling time decreased from about 30 days in the primary tumour and first two transplants to 1·7 days in the tenth transplant. This acceleration was accompanied by a considerable shortening of the mitotic cycle and of its S and $G_1$ phases but without change in the proportion of time spent in S. There was also a reduction in the apparent extent of cell loss and a considerable increase in the growth fraction. Histological changes were noted and studies by feulgen densitometry indicated a considerable shift in ploidy from hyperdiploid to hypertetraploid. The results constitute a detailed example of the effect on tumour growth kinetics of serial transplantation.**

IT is usually recognized that there are many pitfalls in the extrapolation to man of results obtained on tumours in laboratory animals, especially tumours that have been transplanted. Transplantation produces a new tumour growing in a new site and both the site and the tumour cells may differ in important respects from the autochthonous situation (Heiman, 1934). Nevertheless, for reasons of practical convenience, studies of cell population kinetics in neoplastic tissues have largely concentrated on frequently-passaged transplanted tumours (see reviews by Steel, 1970, and by Denekamp, 1970) and a number of authors have attempted to draw conclusions about tumour response to therapy from investigations on tumours that had been passaged many hundreds of times. Such deductions demand a knowledge (or assumptions) about the effects of transplantation on the properties of tumours; the present communication describes a limited study on transplants of one particular experimental tumour which we have followed for a period of 4 years.

### *The BICR/A9 Fibroadenoma*

The tumour, designated BICR/A9, was first observed in December 1967 growing in an untreated non-pregnant breeding female rat of the highly inbred August strain. It presented as a flabby swelling in the right pectoral region. The animal was removed from the breeding room and caged singly for a period of 6 weeks during which caliper measurements were made of the swelling. It grew progressively and when it reached a diameter of 2·7 cm it was transplanted into 6 recipient female August rats. The histological appearance of the primary tumour at this time was characteristic of a fibroadenoma.

Progressive growth occurred in all recipients which were observed with periodic caliper measurements for a period of 240 days. As our interest was in studying the cell population kinetics of tumours whose growth rate was as slow as many human tumours, this tumour was then selected for detailed investigations in its second transplant. Sixty recipients were used, growth occurred in only about half of these (the size of the implanted tissue fragment was probably less than for the first transplant) and the time that the transplants took to reach 0·1 g. varied widely. Animals were selected when the tumours reached 0·1 to 1·0 g. and were killed at various times after single or repeated injections of $^{3}$H-thymidine.

FIG. 1.—Growth curves for the primary tumour and the second, third, fourth and tenth transplants. Tumour weight was estimated by a calibration curve technique (Steel *et al.*, 1966).

During the next two passages there was a considerable acceleration of growth, the transplants grew in all recipients and growth was much more uniform than in the second transplant. In the fourth passage a labelled mitoses experiment was performed on 4 tumours using repeated biopsy.

A larger experiment was again performed in the tenth transplant in which the animals were killed in order to take tumour specimens for the thymidine studies.

*Studies of Tumour Growth*

The tumours in all cases were transplanted subcutaneously into the flank of 6–8 week old syngeneic recipients using a trocar; the acceptance of skin grafts by this strain has been checked within the last 2 years. The tumours were then examined every few days or every 1 or 2 weeks depending on growth rate, with regular measurements of tumour size. For this, each animal was lightly anaesthetized with ether and its tumour measured with vernier calipers in two directions at right angles. The product of these dimensions was used as a measure of tumour size. Values of this product were converted into estimated tumour weights using a calibration curve (Steel, Adams and Barrett, 1966); although the calibration curve was obtained on a different transplanted mammary tumour it was judged that their characteristics were sufficiently similar to justify its use in this way and that this approach was preferable to the use of geometric formulae.

Growth curves for individual tumours were plotted on semilogarithmic paper. In Fig. 1 the mean growth curves are shown, including also that for the third transplant. An interesting feature of these growth curves is that they show little of the upward convexity that is characteristic of many frequently-passaged transplanted tumours over a size of about 1 g. Furthermore, the curves for the second, third and fourth transplants are all *concave* upwards below a size of 0·5 g. A similar observation was reported in our previous description of the acceleration

TABLE I.—*Summary of the Kinetic Parameters for the Primary and Transplanted Tumours*

| | Primary Observed | Second transplant | Fourth transplant | Tenth transplant |
|---|---|---|---|---|
| Date of implantation | Dec. 15, 1967 | Sept. 26, 1968 | Jan. 6, 1970 | Aug. 28, 1970 |
| Time to reach 0·1 g. | — | 205 days | 6 days | 6 days |
| Volume doubling time | 32 days | 30 days | 4·5 days | 1·7 days |
| Labelling index | 1·4% | 5·2% | 15·8% | 31% |
| Duration of $G_2$* | — | 2·5 (2·6; 0·8) | 2·5 (2·6; 0·7) | 2·5 (2·7; 1·0) |
| Duration of S* | — | 17 (19; 10) | 14 (15; 5) | 8·9 (9·2; 2·5) |
| Duration of $G_1$* | — | ~19 (26; 24) | 13 (15; 8) | 3·5 (9·3; 23) |
| Median cycle time (hours) | — | ~41 | 30 | 16 |
| Cell loss factor | — | 68% | 38% | 49% |
| Growth fraction | — | 13% | 38% | 69% |

* Values given are the median durations in hours. Also indicated are the parameters of the lognormal distribution of phase durations given in the form (mean; standard deviation).

of growth of a serially transplanted rat tumour (Steel *et al.*, 1966) where the upward concavity of the growth curves was attributed to the natural selection during the growth of a transplant of cells with the highest potentiality for growth. This conclusion is to some extent supported by the fact that in the data shown in Fig. 1 the initial growth rate of the third transplant is similar to the final growth rate of the second and the initial growth rate of the fourth is similar to the final growth rate of the third.

Estimates of volume doubling time are given in Table I. For the primary tumour this was about 33 days and the estimates for the transplants (made at a size of 1·0 g.) reflect the progressive acceleration to a doubling time of 40 hours in the tenth transplant.

### *Studies of Cell Population Kinetics*

As in a previous communication (Steel *et al.*, 1966) the proliferative state of the tumour cell populations was analysed using a combination of the technique of labelled mitoses (Quastler and Sherman, 1959) and repeated thymidine labelling.

$H^3$-thymidine (Radiochemical Centre, Amersham, catalogue number TRK 61, specific activity greater than 10 Ci/mM) was injected intraperitoneally without anaesthetic, 100 $\mu$Ci per dose. The body weight of recipients was 100–120 g. at the time of transplantation but in the second transplant the range was 150–190 g. by the time of thymidine injection. For repeated labelling, the injections were given at 8-hourly intervals starting at 16.00 hours.

In the second and tenth transplants sufficient tumours were available for animals to be killed in order to take the tumour specimens but in the fourth transplant the labelled mitoses curve was obtained from only 4 tumours by repeated biopsy.

*Tumour biopsy*

The biopsy device consisted of an 18 gauge (1·25 mm. outside diameter) serum needle cut across at right angles to its length, with a sharp cutting edge formed by an internal bevel. The needle was held by a luer mount to an aluminium tube whose opposite end was closed by a disposable skirted rubber vaccine cap. A simple gearing device enabled the needle to be turned at a speed of about 200 rpm by a dental drill and it also brought the axis of revolution of the needle 3 cm. to one side of that of the drill. When the needle was revolving and properly centred a hypodermic syringe with a fine needle and containing saline was pushed into the centre of revolution of the rubber cap. This enabled the revolving needle to be filled with saline and during the cutting operation a negative pressure on the syringe helped to pull tissue into the needle. The biopsy was made through a small skin incision which was later closed by a metal clip. This technique has been found satisfactory for a wide range of types of tumour; with the device clamped, a tumour-bearing rat could be brought on to the needle with one hand and thus it could be operated by one person. Biopsies were made as far apart as possible both in position and in time, not more than 4 being taken from any tumour.

*Autoradiography*

5 $\mu$ paraffin sections were made and coated with Ilford K5 liquid emulsion as described by Lord (1963). The exposure time was 4–6 weeks after which the slides were stained with haematoxylin and eosin. It has been found best to leave autoradiographs unmounted; the optical resolution is thus improved and the slides have not been found to suffer from grain fading.

Slides were examined under oil using a magnification of 1000; the criterion for positive labelling was 4 grains or more. For the labelled mitoses curve only unmistakable mitoses were scored. Only metaphases and anaphases were selected in which individual chromosomes could be discerned. The use of stringent criteria for the selection of mitotic figures reduces the chance of including cells that are abnormal and perhaps degenerating, whilst also in principle improving the resolution of the labelled mitoses curve.

*Analysis of thymidine labelling data*

The data have been analysed on the basis of the model described by Steel *et al.* (1966). The rationale of this approach is as follows. The thymidine labelling data may be affected by many properties of the real tumour cell population on which we have no information. For instance the damping of the labelled mitoses curve is mainly the result of dispersion in the residence time of cells in $G_1$, $G_2$ and S but we do not know the form of the distributions of residence time. We are also ignorant of correlations between the residence times in successive phases and of which cells undergo the transition from proliferation to nonproliferation or to death and loss from the tumour. For such reasons as this, our inferences from the shape of the labelled mitoses curve cannot be absolute. The best course

FIG. 2.—Labelled mitoses curves for the second, fourth and tenth transplants. The full lines are the best-fitting curves found by the method of Steel and Hanes (1971).

of action is to set up a mathematical model which has defined characteristics and to try to find a form of the model which simulates the data. If we succeed, we can then claim to have found plausible values for the various parameters (on the basis of the selected model).

The model of Steel *et al.* (1966) defines the residence time of cells in $G_1$, S and $G_2$ to be independently lognormally distributed. It is a conservative model in which cells once labelled are always labelled, and labelled and unlabelled cells behave identically. Some cells are defined as nonproliferating; they are produced at division with constant probability. The proportion of proliferating cells to total cells (growth fraction) is thus constant with time. Cell loss may occur in various ways and we have defined three forms of the model in this regard:

SAB 1: Only long-lived nonproliferating cells are lost
SAB 2: Cells are lost at or near mitosis
SAB 3: Cell loss is random with respect to age or proliferative state.

These three forms of the model theoretically give different continuous thymidine labelling curves, and it is by examining such curves that we hope to be able to choose the most appropriate form.

FIG. 3.—Repeated labelling curves for the second and tenth transplants. The full lines are theoretical curves calculated on the basis of three models differing with respect to the mode of cell loss (see text).

The analysis of the labelled mitoses data was made using the optimizing computer program described by Steel and Hanes (1971) and the results are shown as full lines in Fig. 2 (see Table I for the corresponding cell cycle parameters). For the second transplant the second peak of the labelled mitoses curve is poorly defined and values for $G_1$ and the whole cycle are therefore imprecise. In view of the small number of experimental points in each curve the values for the standard deviations of the residence times are also imprecise: they are given here merely because these are the parameters that define the shapes of the theoretical curves. Inspection of the three labelled mitoses curves suggests that within the precision of the experimental data the theoretical curves give a satisfactory fit; the chosen model is therefore appropriate (though not unique) for the analyses.

Values for growth fraction and cell loss factor (Steel, 1968) were calculated by the supplementary computer program described by Steel and Hanes (1971). This calculates the form of the age distributions of cells in $G_1$, S, $G_2$ and the whole cell cycle and finds the labelling index of proliferating cells by integration. It also calculates theoretical continuous labelling curves on the basis of the forms of the model described above. These are shown in Fig. 3 together with the data for the second and tenth transplants.

FIG. 4.—Histograms of DNA content per cell for the primary tumour and the second, fourth and tenth transplants.

Repeated labelling was performed at 8-hour intervals. In the second transplant where the median duration of S was 17 hours this implies that all cells entering the S period would be labelled. For the tenth transplant we estimate that less than 5% of cells would be missed at each injection and that the proportion of proliferating cells that could evade repeated injections with this timing would be considerably lower.

EXPLANATION OF PLATE

FIG. 5.—Photomicrographs of Feulgen-stained preparations showing (a) the second, (b) fourth, and (c) tenth transplants. × 600.

Steel, Adams, Hodgett and Janik.

The conclusion to be drawn from the repeated thymidine labelling data (Fig. 3) is that in the second transplant they are consistent with all 3 forms of the model that we have used; in the tenth transplant the data are inconsistent with SAB 1 (loss of long-lived nonproliferating cells) and best support the conclusion that the loss was mainly of proliferating cells.

### *Estimation of Cellular DNA Contents*

The deoxyribonucleic acid (DNA) content of cells from the primary and subsequent transplant generations of the tumour was estimated by Feulgen microspectrophotometry. Tumours were excised immediately after death, fixed in neutral 10% formol-saline and processed by the paraffin method. Sections were cut at 10–12 μm in order to include whole nuclei (Eränkö, 1955) and stained by the Feulgen reaction after cold hydrolysis in 5N HCl. The content of DNA per cell was estimated from the density of the Feulgen staining, measured by a Barr and Stroud integrating microdensitometer (Type GN2) at a wavelength of 550 mμ. The slides were all stained in the same reagents at the same time in order to reduce interslide variations. Lymphocytes and myelocytes within the tumour were also measured to establish the basic diploid value; between different slides and different tumours the estimates of diploid value ranged from 6·4 to 6·7 arbitrary units and the mean of 6·5 was taken as the actual diploid value.

Measurements of DNA content do not directly indicate the chromosome ploidy distribution in proliferating cell systems but the changes seen in successive transplants of the present tumour are large enough to establish a gradual shift in chromosome number from hyperdiploid to hypertetraploid. The primary tumour (Fig 4) had a clear mode in the 3N (triploid) region and content varied from 2N to 4N amounts of DNA. In the second transplant the distribution was similar but with a slight increase in the average DNA content. In the fourth transplant there was a marked change, with a mode in the 4N (tetraploid) region and a range of DNA content up to above 8N (octaploid). In the tenth transplant this trend was continued; on the size of sample that it was possible to take there was no clear mode but a range of DNA content from 4N to above 16N.

Other histological changes were also noted. The cell density (nuclei per unit area of section) and mean nuclear diameter both increased, particularly between the second and fourth transplants (Fig. 5). The primary tumour contained a large proportion (estimated at 25%) of apparently normal fibroblasts, lymphocytes and polymorphs. This proportion decreased to perhaps 3% in the tenth transplant. The primary tumour was very rich in mast cells and the proportion of these fell considerably with successive transplantation. The histological changes associated with serial transplantation of benign neoplasms of rat mammary tissue were described by Heiman (1934). He observed great differences from one primary tumour to another in the speed and character of the transformation that occurs under repeated passaging; the changes in the present tumour correspond with his observations of sarcomatous transformation.

### DISCUSSION

Serial transplantation of a tumour provides an opportunity for cell selection. The total growth of any one transplant from a fragment weighing a few milligrams to a tumour weighing a few grams involves about 10 doublings of cell number and because of cell loss and the presumed existence of nonproliferating cells, the proliferating cells must go through a number of generations during the course of each doubling. For the second, fourth and tenth transplants of BICR/A9, this number was respectively about 17, 4 and 3. The total number of cell generations that are produced during the complete growth of a transplant is thus large and for the early transplants probably in the region of 100.

In each generation there is the opportunity for cell selection with respect to various cellular characteristics and in so far as these are capable of being transmitted from parent to daughter cells, there will be a progressive selection of cells that have the greatest potentiality for rapid growth. This selection may not only be for short intermitotic time but also for such properties as ability to grow under conditions of poor nutrient supply. The acceleration of growth observed in this and other experimental tumours (McCredie *et al.*, 1971) is a reflection of such cell selection and adaptation. Within the first 10 transplant generations the volume doubling time decreased to almost one-twentieth of the doubling time of the primary tumour. There was little change by the second transplant but then a marked change between the second and fourth transplants. The estimates of DNA content (Fig. 4) showed similar variations, with little change between the primary and second transplant but a much greater trend towards polyploidy by the fourth transplant generation. These changes might be linked to the operation of an immunological selection process (Janik, 1971) whose existence is suggested by the initially high proportion of mast cells. Similar changes in ploidy were observed by Dux *et al.* (1967) in studies of Ehrlich ascites tumour transplanted into rats.

The timing of the mitotic cycle was investigated in the second, fourth and tenth transplants. The results (Table I) showed that the $G_2$ period did not change but that there was a progressive shortening of the S and $G_1$ periods. These phases changed to the same extent: thus the ratios of the mean S phase duration to the mean cycle time in the three transplants were 0·41, 0·46 and 0·43. The level to which a labelled mitoses curve damps out should theoretically also give this ratio, and in fact these levels were statistically indistinguishable. This result is reminiscent of the observation of Steel (1970), that published labelled mitoses curves for primary C3H mammary tumours (Mendelsohn, 1965) and for the first generation transplants of these tumours (Denekamp, 1970) differ only in time-scale.

The thymidine labelling index increased between the primary tumour and the tenth transplant in inverse proportion to the decrease in volume doubling time. Estimates of cell loss factor (Steel, 1968) could only be made on the transplants; such estimates are always very approximate (Owen and Steel, 1969) and it can only be concluded that beyond the second transplant there was a reduction in cell loss factor from about 70% to about 40%. Estimates of growth fraction are usually similarly imprecise because of the uncertainty of our knowledge of the labelling index of proliferating cells. This index is close to the ratio of the mean S period to the mean duration of the intermitotic period which, as mentioned above, seems rather constant between the three transplant generations. The increase in the calculated values for growth fraction from 13% in the second to about 70% in the tenth transplant should therefore be reliable.

At the present time the relationship between the kinetics of cell proliferation in a tumour and its response to radiotherapy or chemotherapy is not clear.

Probably the main factor which prevents this relationship from being understood is the likelihood that the cells that have the capacity to regrow a tumour after treatment form only a small proportion of the whole cell population of the tumour (Mendelsohn, 1967; Bush, 1970). Autoradiographic studies give information about the cell population taken as a whole; to understand therapeutic response we need information specifically on the clonogenic cells. It is not known how the selection imposed by transplantation operates on the proportion and proliferative characteristics of clonogenic cells. It could be that repeated transplantation selects for a higher clonogenic fraction and that clonogenic cells in the primary tumour have a cell cycle which resembles that found in the later transplants. This work is now being extended to include studies of the clonogenic cells.

We acknowledge with gratitude the support and encouragement of Professor L. F. Lamerton and the technical assistance of Mrs. J. Lucas.

### REFERENCES

Bush, R. S.—(1970) *Brookhaven National Laboratory Publications* 50203 (C-57), p. 151.

Denekamp, J.—(1970) *Cancer Res.*, **30**, 393.

Dux, K., Bregula, U., Slowikowska, G., Lisowska, B. and Jagora, K.—(1967) U.I.C.C. Monograph Series Vol. 2, pp. 351–360. Edited by R. J. C. Harris. Copenhagen (Munksgaard).

Eränkö, O.—(1955) ' Quantitative Methods in Histology and Microscopic Histochemistry '. Basel (S. Karger).

Heiman, J.—(1934) *Am. J. Cancer*, **22**, 497.

Janik, P.—(1971) *Cell & Tissue Kinetics*, **4**, 69.

Lord, B. I.—(1963) *J. photogr. Sci.*, **11**, 342.

McCredie, J. A., Inch, R. and Sutherland, R. M.—(1971) *Cancer*, **27**, 635.

Mendelsohn, M. L.—(1965) ' Cellular Radiation Biology '. University of Texas, M. D. Anderson Hospital. (Williams & Wilkins). pp. 498–513.—(1967) ' Radiation Research ', edited by G. Silini. Amsterdam (North Holland).

Owen, L. N. and Steel, G. G.—(1969) *Br. J. Cancer*, **23**, 493.

Quastler, H. and Sherman, F. G.—(1959) *Expl Cell Res.*, **17**, 420.

Steel, G. G.—(1968) *Cell & Tissue Kinetics*, **1**, 193.—(1970) *Brookhaven National Laboratory Publications* 50203 (C-57).

Steel, G. G., Adams, K. and Barrett, J. C.—(1966) *Br. J. Cancer*, **20**, 784.

Steel, G. G. and Hanes, S.—(1971) *Cell & Tissue Kinetics*, **4**, 93.